# A system to rapidly develop a bunyavirus pseudotyped virus neutralisation assay for pandemic preparedness

**DOI:** 10.1038/s41541-025-01278-8

**Published:** 2025-11-06

**Authors:** Federica Marchesin, Emma M. Bentley, Francis Mutuku, Victor Jeza, Bryson Ndenga, Sarah Kempster, Stuart D. Dowall, Neil Almond, Edward Wright, Ashley C. Banyard, Hubert Buczkowski, Nazif Elaldi, Ali Mirazimi, Roger Hewson, Nicola J. Rose, Yasuhiro Takeuchi, Giada Mattiuzzo

**Affiliations:** 1 Medicines and Healthcare products Regulatory Agency, South Mimms, UK; 2 University College London, London, UK; 3 Technical University of Mombasa, Mombasa, Kenya; 4 Kenya Medical Research Institute, Nairobi, Kenya; 5 UK Health Security Agency (UKHSA), Porton Down, UK; 6 University of Sussex, Falmer, UK; 7 Animal and Plant Health Agency, Addlestone, UK; 8 Sivas Cumhuriyet University, Sivas, Turkey; 9 Public Health Agency of Sweden, Solna, Sweden; 10 Karolinska Institute, Stockholm, Sweden; 11 Swedish Veterinary Agency, Uppsala, Sweden; 12 London School of Hygiene and Tropical Medicine, London, UK

**Keywords:** Biotechnology, Microbiology

## Abstract

Several *Bunyavirales* families have been listed as being of high pandemic or epidemic risk by the WHO R&D Blueprint. To support pandemic preparedness and the 100 Days Mission, along with rapid provision of vaccines and therapeutics, the development of tools to assess the immune response is required. Pseudotyped viruses (PV) have been shown to be a suitable alternative to authentic infectious virus to measure virus-neutralising activity, a key component of the immune response. They alleviate the need to acquire and amplify viral isolates and do not require high containment facilities. Generating PV of some families within the class *Bunyaviricetes* is challenging because of a lack of co-localisation of viral glycoproteins at the vector budding site. Here, we describe a versatile plug-and-play system focusing on two prototype viruses for the family *Phenuiviridae*, Rift Valley fever virus (RVFV) and for the family *Nairoviridae*, Crimean-Congo haemorrhagic fever virus (CCHFV). Shared key parameters for the production of RVFV and CCHFV PV were identified and optimised on a single-cycle, recombinant vesicular stomatitis virus vector (VSV), which allowed for the successful and rapid production of PV for Dabie bandavirus and Oropouche virus. We propose that this system could be successfully applied to other high-consequence bunyaviruses, including those yet unknown, which may emerge in the future. Assessment of the novel bunyavirus PV generated here demonstrated a good correlation with traditional neutralisation assays with infectious virus. This system offers an adaptable and widely accessible platform that can be rapidly developed in response to emerging viral threats.

## Introduction

The declaration of a Public Health Emergency of International Concern (PHEIC) is the WHO’s highest alert level, implemented under International Health Regulations (2005)^1^. It was defined to address the need for a rapid and global response to counter disease outbreaks constituting a public health risk. In the last 20 years, there have been eight PHEIC declarations, which have increasingly highlighted the importance of initiatives for pandemic and epidemic preparedness, including the 100 Days Mission^2^. Medical countermeasures (MCM) are required to be effectively prepared to contain future outbreaks, such as readily available vaccines and therapeutics. The WHO Research and Development (R&D) Blueprint for Epidemics has recently produced a list of families of pathogens with high potential to cause a PHEIC^3^. Most of these diseases are caused by hazard group 3 and 4 pathogens, which can affect the speed of MCM research. Serological assays, using infectious virus, are often considered the gold-standard method to assess the humoral responses elicited by vaccines or the potency of therapeutics. However, this step is often low through-put, expensive and time-consuming when the infectious virus must be handled in a high containment level laboratory.

The use of pseudotyped viruses (PV), that are viruses genetically engineered as vectors that can display on their surface glycoproteins of a heterologous virus of interest, offers a safer and rapid alternative to handling of the authentic virus^4,5^. Usually, the gene coding for the glycoprotein of the viral vector is replaced with a reporter gene to facilitate the detection of viral entry, which is mediated by the glycoprotein of the heterologous virus. Widely used viruses to generate PV are based on the core of enveloped viruses such as murine leukaemia virus, human immunodeficiency virus (HIV) and vesicular stomatitis virus (VSV)^5–7^. The role of PVs during outbreaks became evident during the COVID-19 pandemic, where they were crucial in the early identification of the SARS-CoV-2 receptor^8,9^ and in neutralisation assays to monitor immune responses to infection and vaccination^4,10–12^.

Production of PV has been successfully conducted for several high-consequence viruses, including members of the Filovirus, Coronavirus and Bunyavirus families^13–17^, among others. However, the class *Bunyaviricetes* includes families for which PV generation is challenging and requires optimisation^18^. This is due to the viral replication cycle involving assembly and budding from the intracellular membrane system, such as the Golgi apparatus^19^. The glycoproteins of these viruses are therefore located in the cell organelles, while the budding and acquisition of the glycoproteins for many vector systems occurs at the cell membrane^20,21^. The different localisation between heterologous glycoproteins and PV core affects the efficiency of viral particle production. Yet successful PV production has been reported for some Golgi-budding viruses, suggesting envelope proteins may localise on the cell surface as well, although the procedure may require time-consuming optimisation^22–28^.

Here, we have identified the parameters for a systematic approach to quickly and reproducibly generate PV for bunyaviruses using two prototype viruses of the *Bunyaviricetes* class, Rift Valley fever virus (RVFV) of the family *Phenuiviridae* and Crimean-Congo haemorrhagic fever virus (CCHFV) of the family *Nairoviridae*. Using a VSV-based vector, the same conditions have been applied to successfully pseudotype the glycoproteins of other bunyaviruses of public health interest, such as Dabie bandavirus (DABV, family *Phenuiviridae*), the aetiological agent for the Severe Fever with Thrombocytopenia Syndrome and Oropouche virus (OROV, family *Peribunyaviridae*). Both viruses are listed in the WHO R&D Blueprint as prototype viruses within their families, with the potential for PHEIC as high for DABV and low for OROV^3^. However, an increase in the number of Oropouche fever cases since December 2023, including outside endemic regions and with more severe outcomes, has raised public health concerns^29^.

We have further confirmed the utility of these bunyavirus PV in serological assays by comparison to the authentic virus neutralisation test. The pseudotyping system described here allows for the rapid generation of PV, which can be used to respond in a timely manner to an outbreak of a known or unknown but related bunyavirus and used as a safer, more convenient alternative to the authentic virus in serological assays for the development and validation of MCM.

## Results

### VSV is a better platform for the pseudotyping of bunyaviruses

**Fig. 1:**
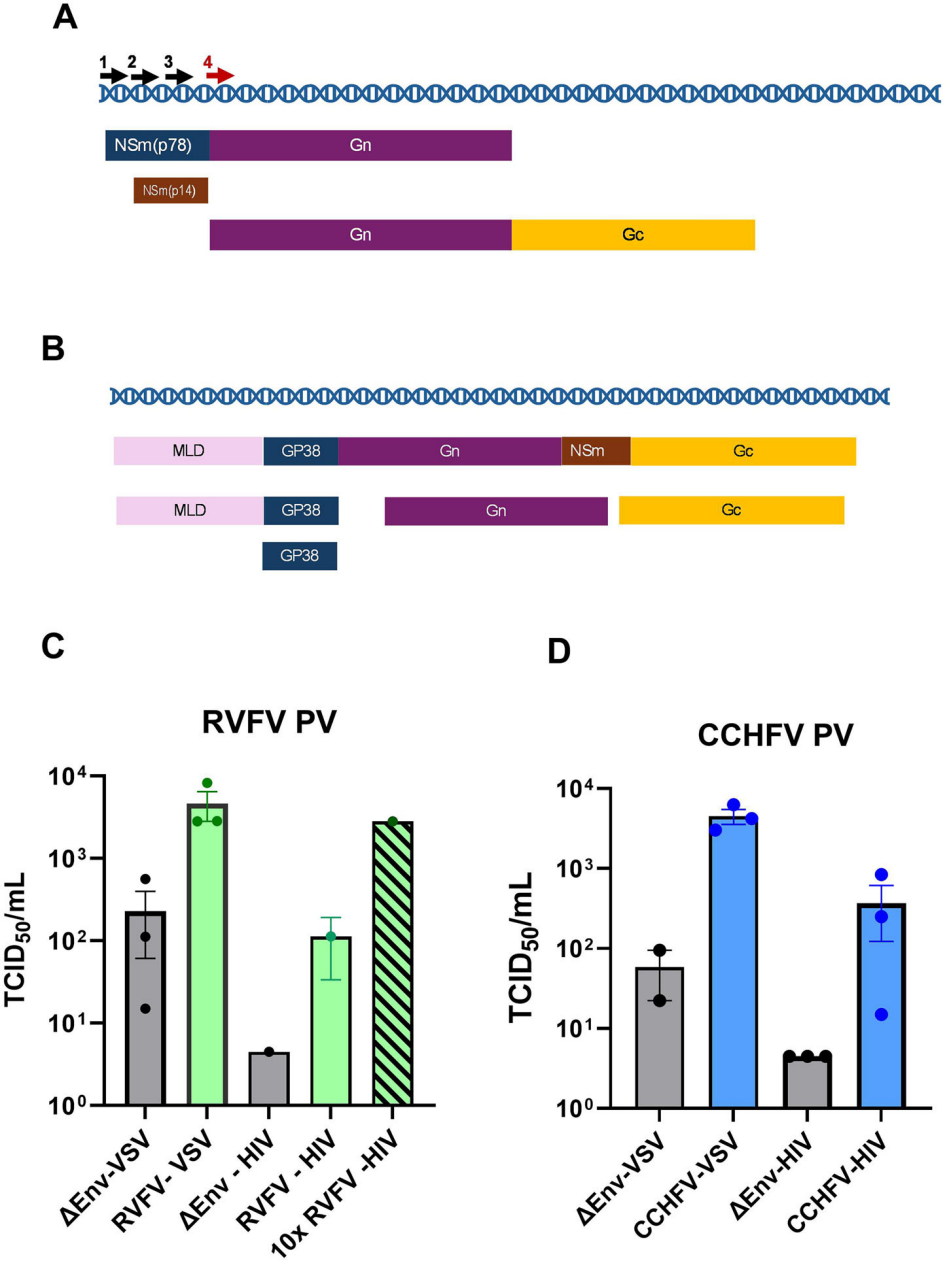
Comparison of platforms for generating bunyavirus PV. RVFV (**A**) and CCHFV (**B**) M segment in open reading frame expression, with arrow indicating the four potential RVFV GnGc start codons. MLD mucin-like domain. Recombinant VSV or HIV-1-based vectors expressing the firefly luciferase gene were used to generate PV for RVFV (**C**) or CCHFV (**D**). The PV was titrated on Vero cells (VSV-based) or CRFK cells (HIV-1-based) and titres expressed as 50% tissue culture infectious dose (TCID_50_) per mL determined by the detection of luminescence and using Spearman–Karber formula. The graph shows the mean value of three independent experiments ± standard error of the mean.

For the production of RVFV PV, we compared two systems which have historically been successfully used, the non-replicative ΔG-VSV and a lentiviral vector based on HIV-1^28,30^. The RVFV polyprotein GnGc precursor is encoded by the Medium (M) segment of the viral genome and is processed into the Gn and Gc components, which form a disulphide-linked heterodimer^31^. There are four in-frame starting codons in the M segment for the translation of the polyprotein GnGc, with the fourth in-frame ATG being suggested as the most efficient start codon for expression^32^ (Fig. 1A). For CCHFV the whole M segment sequence was used for PV production, as the role of other M-coded proteins (GP38 and NSm) have been shown to be important for CCHFV particle production^33^ (Fig. 1B). RVFV full-length GnGc, starting at the fourth in-frame start codon and CCHFV GnGc sequences were synthetised and cloned into the pI.18 expression plasmid and used in the production of PV following existing in-house protocols for the production of PV. For RVFV, the yield using the VSV was approximately ten-fold greater than HIV-1, and the lentiviral system could only achieve comparable titres following a tenfold concentration by ultracentrifugation (Fig. 1C). Similar results were observed for the production of CCHFV PV (Fig. 1D), suggesting that, without optimisation, the VSV-based vector is the best candidate for the production of RVFV and CCHFV PVs.

### Shared optimal conditions for RVFV and CCHFV VSV-based PV production

**Fig. 2:**
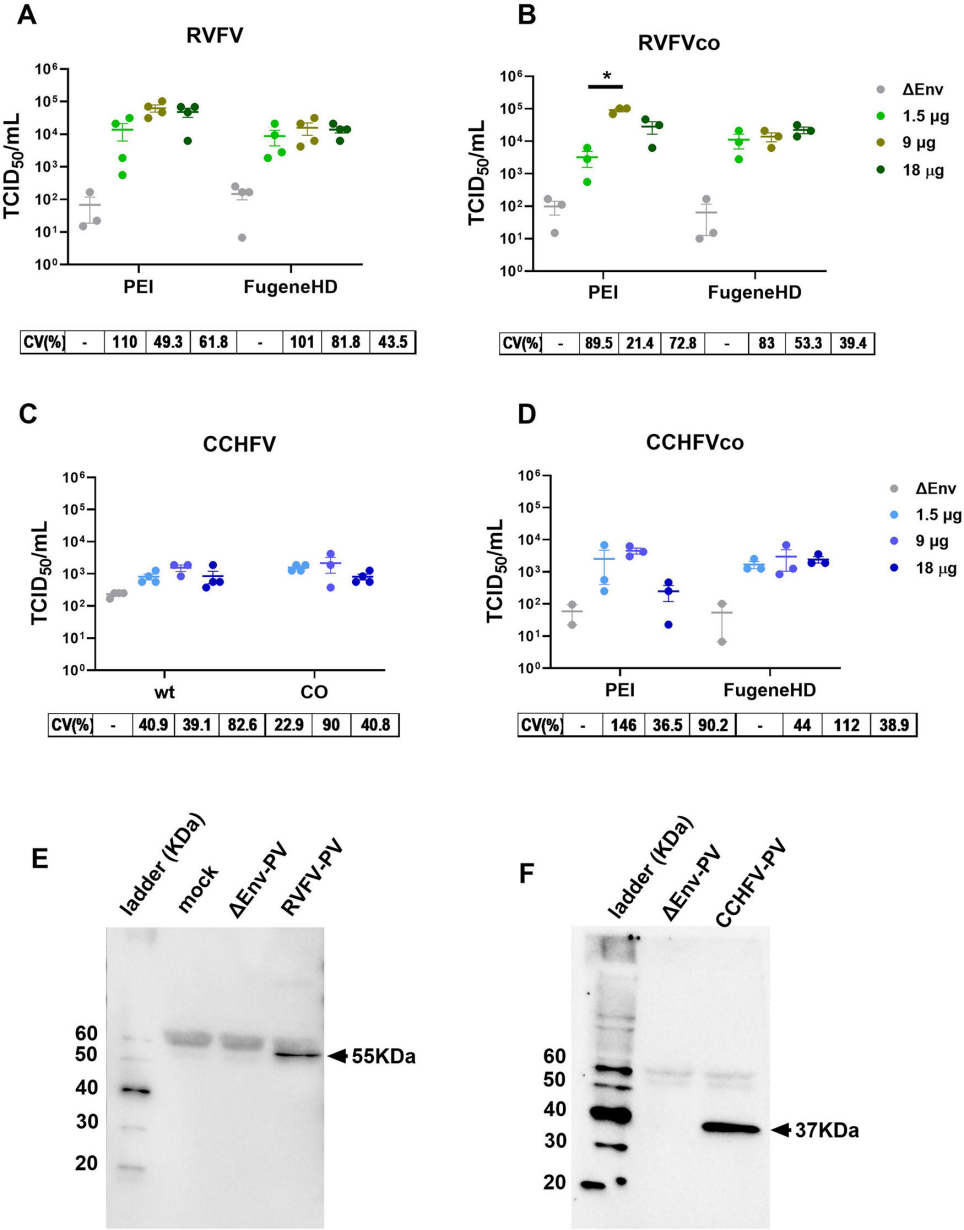
Identification of key parameters for the production of bunyavirus PV. Increasing amounts of expression plasmid for the native sequence of RVFV GnGc glycoproteins (**A**) or human-codon optimised sequences (**B**) were used to transfect HEK293T/17 cells using either PEI or FugeneHD to produce VSV-based PV. Comparison of the original (wt) against codon optimised (CO) CCHFV GnGc sequence for the production of VSV-based PV using PEI transfection reagent and increasing amount of expression plasmid (**C**). CCHFV-PV titres using the codon-optimised GnGc sequence were compared between the two transfection reagents (**D**). Titres are reported as 50% tissue culture infectious dose (TCID_50_) per mL. Results for three or four independent productions are reported as mean titre ± standard error of the mean. The coefficient of variation (CV%) is reported below each graph. Immunoblot of the VSV-based pseudotyped particles using anti-RVFV Gn (**E**) or anti-CCHFV Gn (**F**) monoclonal antibodies. The predicted size of the monomer is indicated in the blot.

Selected parameters during the production of RVFV and CCHFV VSV-based PV were evaluated to determine which are key to obtain the highest and most reproducible PV titre. Factors that could affect the expression level of GnGc and its availability at the cell surface were assessed, as these are critical for the incorporation into the VSV-based PV particles. Therefore, the transfection of the human embryonic kidney (HEK)293T/17 producer cells was optimised to overexpress GnGc prior to infection with the recombinant G-VSVΔG-Luc virus. Increasing amounts of pI.18_GnGc plasmid were used in combination with two types of transfection reagent, a mixed lipid-based, FuGENE HD, and a cationic polymer-based, polyethylenimine (PEI) (Supplementary Fig. 1). Another key parameter investigated was the effect of using human-codon optimised GnGc sequences for the transfection of the producer HEK293T/17 cells, as it has been reported that may impact protein expression^34^ (Fig. 2A–D, Supplementary Fig. 1). For RVFV-PV, when using FugeneHD as transfection reagent, there was not a statistically significant difference between amounts of GnGc plasmid used and the resulting PV titre (Fig. 2A, B). The only statistically significant difference was observed between 1.5 μg and 9 μg using PEI in combination with codon optimised RVFV GnGc (*p* = 0.0101 by one-way ANOVA, Fig. 2B). Indeed, 9 μg of plasmid used to transfect 4 million HEK293T/17 cells, seeded 24 h ahead, in combination with a PEI transfection reagent, achieved the highest PV titres with the lowest standard deviation between independent productions for both RVFV and CCHFV (Fig. 2B, D). Although no statistically significant increase in PV titre was observed using human-codon optimised GnGc sequences, there was a trend towards higher yield with the codon optimised sequences. To confirm the incorporation of the glycoproteins on the PV, RVFV and CCHFV Gn proteins were detected by immunoblotting of the PV-containing supernatant from the producer cells (Fig. 2E, F).

### Application of the optimised bunyavirus PV system showed a different cell line sensitivity for other members of bunyavirus families

**Fig. 3:**
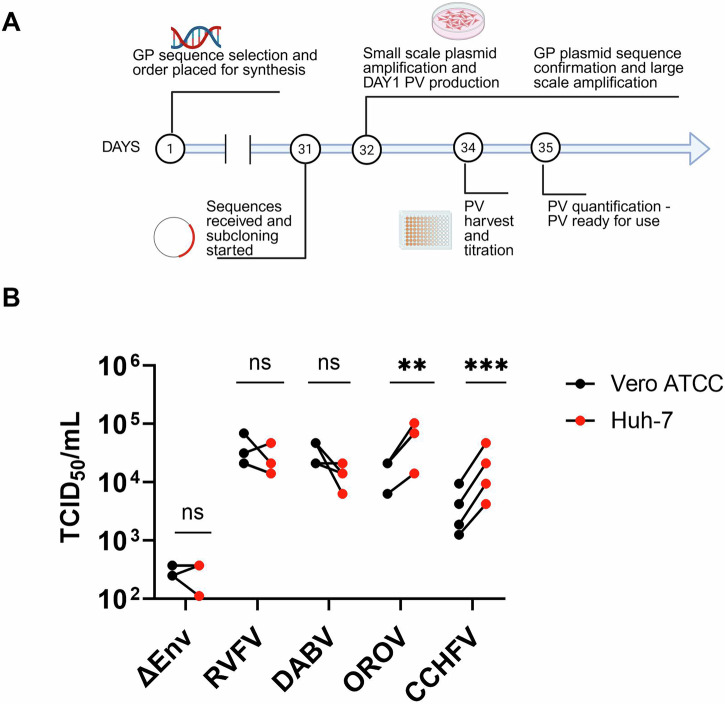
Plug-and-play system for bunyavirus PV production can identify optimal target cells. Schematic of the timeline for the production of functional PVs once the viral sequence is published and assessment of usable PV. Created in BioRender https://BioRender.com/9cj39l1 (**A**). Human-codon optimised sequences of Rift Valley fever virus, (RVFV), Dabie bandavirus (BADV), Oropouche virus (OROV) and Crimean-Congo haemorrhagic fever virus (CCHFV) GnGc sequences were used to produce PV and titrated on Vero (black) or Huh-7 (red) cells (**B**). PV titres are reported based on luciferase activity as TCID_50_/mL. Each point indicates an independent production of the PVs. The significance between the titration on Vero *versus* Huh7 was determined by a two-tailed paired t-test on log-transformed TCID_50_/mL titres. ** *p* = 0.005; ***p = 0.0005.

The system developed for the production of RVFV and CCHFV VSV-based PV was tested for other bunyaviruses, namely Dabie bandavirus (DABV), which is classified alongside RVFV in the family *Phenuiviridae*, and Oropouche virus (OROV), family *Peribunyaviridae*. Human-codon optimised GnGc sequences were synthesised and cloned within the same pI.18 expression plasmid used for RVFV and CCHFV PV generation. Upon receipt of the synthetised GnGc sequences, DABV and OROV-PV were generated at usable titre within 5 days (Fig. 3A, days 31–35). Another key parameter in the setup of this system is the choice of target cells for downstream applications. For the titration of the bunyaviral PV, we selected two cell lines, which are commonly used for bunyavirus infectivity assays, monkey Vero cells and a human hepatocyte-derived Huh7 cell. Other cell lines were also tested by the RVFV/VSV PV confirming that Vero and Huh7 are a suitable choice, albeit titration on Vero E6 produced similar results (Supplementary Fig. 2). Using the conditions identified for the successful production of RVFV and CCHFV VSV/PV, it was possible to produce DABV and OROV PV without the need of optimisation, with titres comparable to RVFV (Fig. 3B). PV titres for both viruses within the *Phenuiviridae* family (RVFV and DABV) were similar between Vero and Huh7 cells, while CCHFV and OROV-PV titres were statistically higher in Huh7 cells. This is likely to be reflective of a difference in receptor usage between the virus families.

### Antibodies specifically neutralise the respective bunyavirus PV

**Fig. 4:**
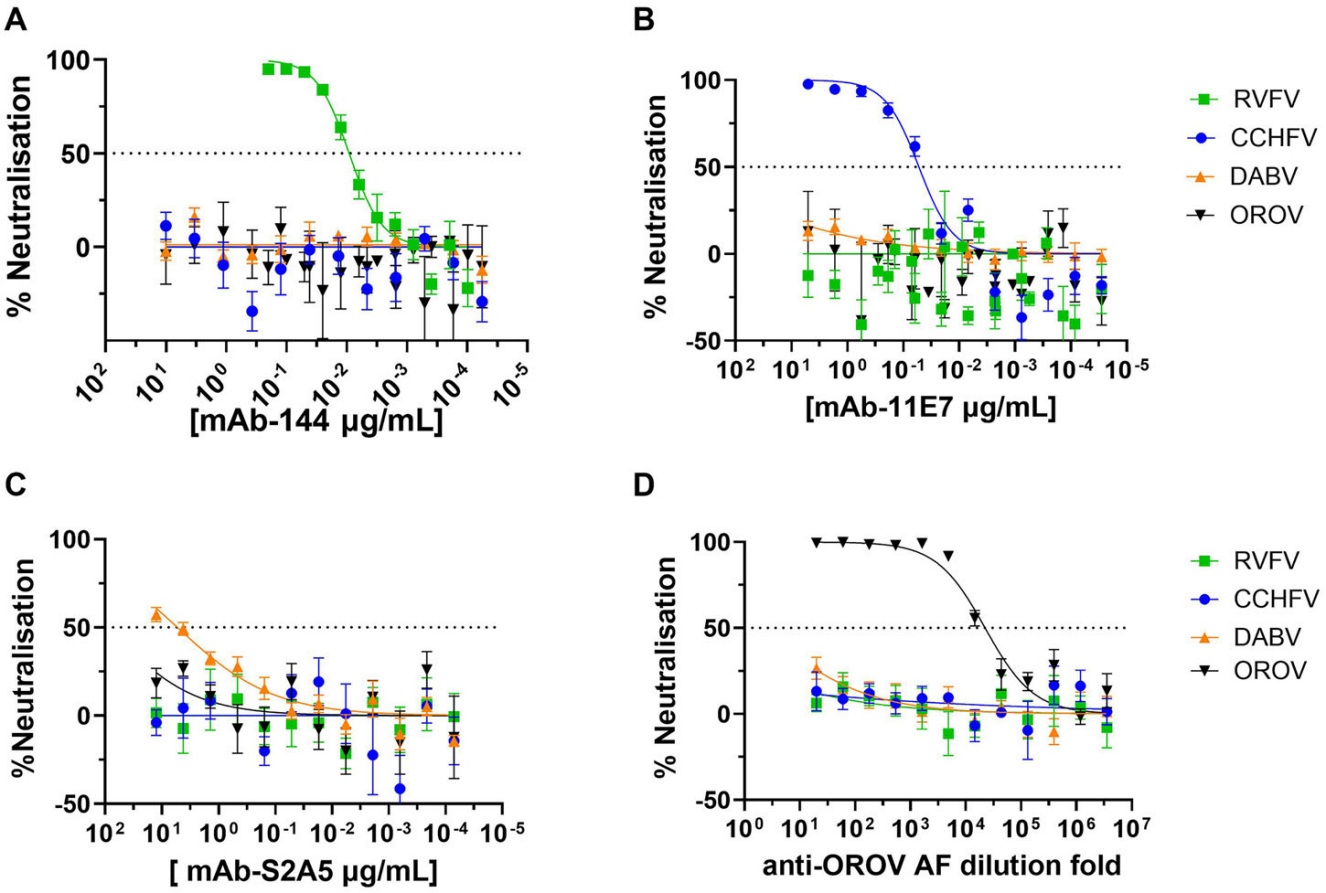
Specific antibodies neutralisation of the respective PV. 100 TCID_50_ of RVFV (green) CCHFV (blue) DABV (orange) or OROV (black) VSV-based PV were incubated with serial dilutions of anti-RVFV mAb-144 (**A**), anti-CCHFV mAB-11E7 (**B**), anti-DABV S2A5 (**C**) or anti-OROV ascitic fluid ATCC VR1228AF (**D**), prior to addition to Huh7 cells. Percentage of neutralisation was calculated based on luciferase activity relative to PV only. The graphs show average values ± standard error of the mean of at least two experiments conducted in duplicate.

The bunyavirus PV generated was tested in neutralisation assays against commercially available antibodies (Ab) raised against each specific virus. Three known monoclonal antibodies for RVFV (mAb-144)^35^, CCHFV (mAb-11E7)^36^, DABV (mAb-S2A5)^37^ and a polyclonal mouse anti-OROV ascitic fluid (ATCC VR1228AF)^38^ were titrated against the four PVs. A dose response curve with a reproducible neutralisation titre was only obtained by each Ab against the homologous virus, whilst against the other PVs, the Ab failed to achieve a percentage of neutralisation above 50% (Fig. 4). The lower neutralising potency of mAb-A2A5 against DABV PV (Fig. 4C) was expected against the isolate 14KS25 (genotype B)^37^. These results demonstrated that the titre of bunyavirus PV generated in our system is suitable for downstream applications, such as neutralisation tests, and that the antibody responses are largely specific for each bunyavirus PV.

### Bunyavirus PV neutralisation is comparable to authentic virus neutralisation

**Fig. 5:**
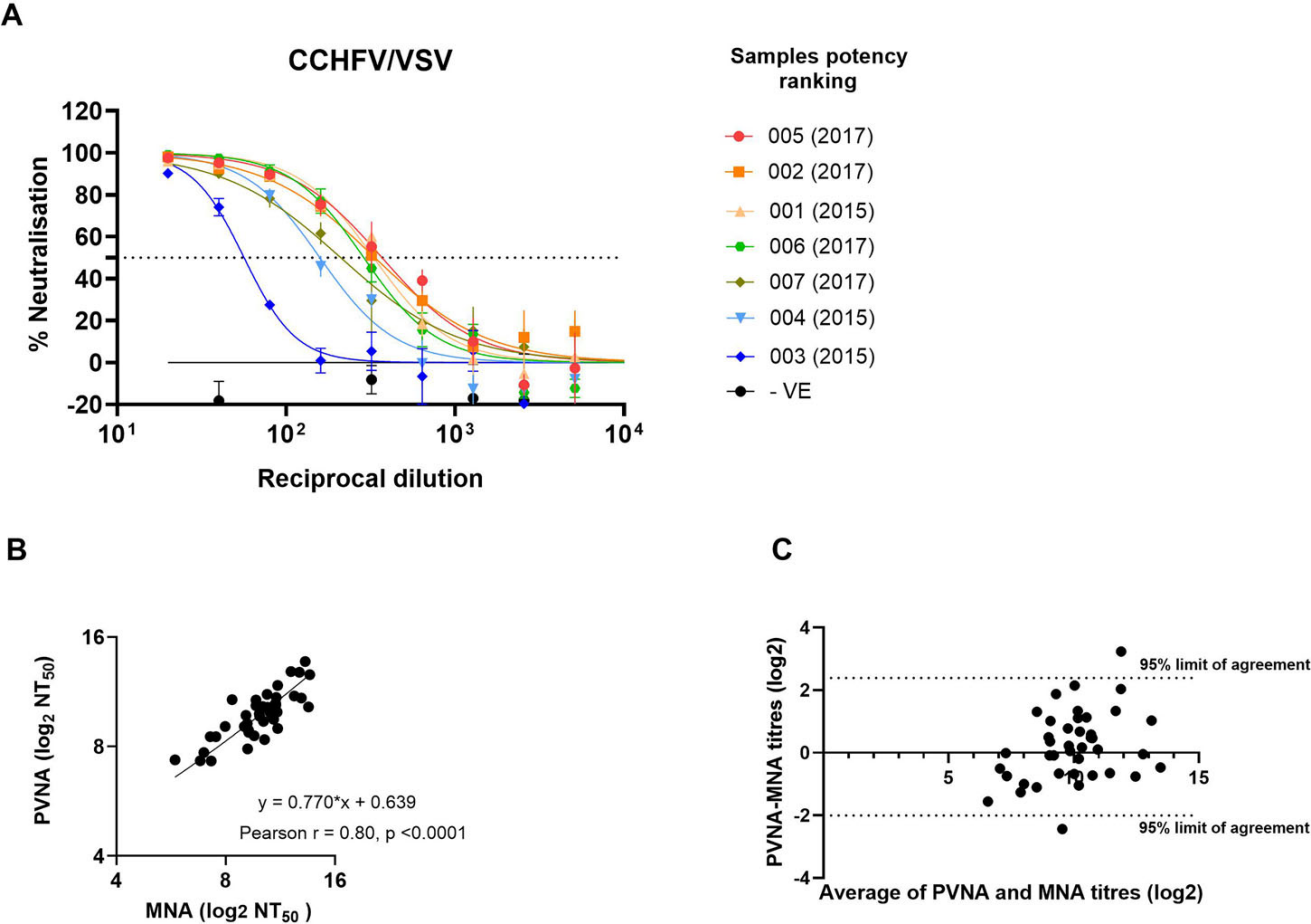
PV-based neutralisation test on convalescent samples and correlation with authentic virus neutralisation assay. Plasma from eight donors, seven CCHF convalescent and a negative (−VE) healthy individual, was tested against CCHFV/VSV PV on Huh7 cells. Percentage of neutralisation was calculated based on luciferase activity relative to PV only. The graphs show average values ± standard error of the mean of two experiments conducted in duplicate. Samples were ranked from the highest (top) neutraliser to the lowest (**A**). 60 serum samples from Kenya were tested in a microneutralisation assay using RVFV ZH501 and in a RVFV (ZH548)/VSV PV on Vero cells. For the 41 positive samples, neutralisation titres expressed as 50% neutralisation titre (NT_50_) were calculated using a 4-parameter regression analysis and log_2_-transformed data. Titres were compared through Deming regression (**B**) and Bland-Altman model (**C**) using GraphPad Prism version 10.3.1.

The use of PV in neutralisation assays as an alternative to the authentic virus has previously been reported for both RVFV/VSV PV and for CCHFV/VSV PV^39,40^. To confirm the suitability of the PVs as an alternative to the authentic virus, we tested plasma from seven individuals with a known past CCHFV infection and compared the results with previously published work on the same samples^41^. All the samples produced a dose-response curve against the CCHFV-PV and, with the exception of sample 001 (2015), the ranking of the remaining samples correlated well with the reported data (Fig. 5A). Specifically, samples 005 and 002 were the highest titre as shown in the CCHFV authentic virus neutralisation assays^41^. Ranking of the samples in the published work^41^ differed between methods used; therefore, it is not surprising that sample 001 was not detected as positive by the authentic CCHFV, but was positive in the PVNA. We further looked at the correlation between the neutralisation results obtained using the PV-based neutralisation test against the authentic virus neutralisation assay for RVFV/VSV PV. A panel of 60 serum samples from individuals with known or suspected exposure to RVFV were tested by PV neutralisation assay (PVNA) with RVFV/VSV PV and by micro-neutralisation assay (MNA) with RVFV isolate ZH501 (Fig. 5B). Among those samples, 41 showed neutralising activity by both the MNA and the PVNA, with the remaining samples testing negative by both assays. When comparing the results from the MNA and the PVNA, the Pearson correlation test showed a statistically significant (*p* < 0.0001) positive correlation (*r* = 0.8027, 95% confidence interval 0.6574–0.8905) between the two measurements, which was confirmed by Deming regression analysis with a slope of 0.7699, and an intercept of 0.6386 indicating slightly higher antibody titre values for the PVNA (Fig. 5B). To test the agreement between the two methods, a Bland-Altman agreement test was performed demonstrating a high degree of comparability with a difference (bias) of 0.1948 and only 2 out of 41 positive samples outside the 95% limits of agreement (Fig. 5C).

## Discussion

The COVID-19 pandemic was one of the first examples of a Disease X outbreak scenario since the concept was first introduced in the 2018 revision of the WHO R&D Blueprint^42^. The response to the pandemic was facilitated by the knowledge acquired during the previous outbreaks of other coronaviruses in the same family (SARS-CoV and MERS-CoV). This supports the most recent concept of listing priority families of pathogens for the research and development of MCM to enhance preparedness for known and unknown threats^3,43^. During the COVID-19 pandemic, the use of PV for SARS-CoV-2 variants, was critical to investigate host–pathogen interactions, such as receptor usage^9^, immune evasion^44,45^, vaccines^11,12^, therapeutics^46,47^ and for serosurveillance^48^. These important tools represent a useful instrument for a rapid response to viral outbreaks. Most of the platforms to generate PV are based on viruses which exit cells from the cell membrane, acquiring the viral glycoproteins of the virus of interest during this step^5,6,20^. Viruses that assemble in the intracellular membrane system, such as the endoplasmic reticulum and Golgi apparatus, represent a challenge to pseudotyping^49^. Within the bunyaviral family, viruses have a cell cycle with assembly and budding taking place within the intracellular membrane system, such as the Golgi apparatus, where the glycoproteins Gn and Gc are retained^19^. In this work, we have developed a system to generate bunyavirus PVs and showed that it is suitable for the rapid production of PV for four bunyaviruses from three distinct families.

The key parameters identified and optimised for RVFV and CCHFV allowed for the generation of DABV and OROV PV with titres suitable for serological assays within 5 days from receipt of the synthesised GnGc sequences. This timeframe includes cloning into a suitable expression vector and titration on cell lines which are likely to support infectivity of these viruses. The choice of target cell line for the infectivity/neutralisation assay is also critical. As most of the cell receptors for the prototype viruses in each bunyavirus family are known, looking at the expression of these proteins in candidate target cells offers a good starting point. For instance, the CCHFV-PV reproducibly produced a significantly higher PV titre on Huh7 cells than Vero cells (Fig. 3B); this fits with published evidence that the CCHFV entry factor, low-density lipoprotein receptor, has higher expression in Huh7 than Vero cells^50^. However, this is a simplistic approach, and the entry process often involves more than one entry factor. Indeed, both RVFV and OROV share an entry factor, low-density lipoprotein-related protein 1 (Lrp-1)^51,52^, but OROV shows a significantly higher titre on Huh7 than Vero cells, while RVFV does not (Fig. 3B). Increasing knowledge on receptor usage by viruses in the same or related families is critical for the choice of cell line for implementing an infectivity assay in a timely manner for a newly discovered related virus. Some candidate cell lines, such as Huh7 and Vero cells, will be key for initial evaluation of other bunyavirus families in the context of serological assays, but other more physiologically relevant cell lines could be more appropriate for other studies.

Other key parameters identified while setting up this pseudotyping system target the expression of the bunyavirus glycoproteins in the PV-producer cells, specifically the amount of GnGc expression plasmid and transfection reagent. Codon optimisation to increase expression in the producer cell line did not significantly impact the PV production, although there is a pattern towards a higher titre (Fig. 2A–D). This suggests that codon-optimisation for mammalian expression of the bunyavirus glycoprotein sequences, which predominantly circulate between arthropods and humans, could have a neutral or beneficial impact and is in contrast with that reported for other viruses^53^. We did not investigate extensively other parameters in the VSV-based PV production after the infection with the recombinant ΔG-VSV-Luc vector, as these are more conserved regardless of the virus of interest^22,23,26,27,30^ and generally follow the original protocol as described previously^6^.

Although the PV-based methods have been adopted by several laboratories and networks^8–10,28,54^, there is still a need to demonstrate correlation with the results obtained with the authentic virus as shown here (Fig. 5). Following optimisation of production and confirmation of correlation with authentic virus, the additional quality control of each batch of PV becomes an important step. While for some applications, such as investigation of virus-host interactions, variation between each batch may not have a critical impact, for others, such as serological assays, reproducibility in the characteristics of the PVs plays a critical role. The evidence supporting the implementation of PV as a safer and rapid alternative to authentic virus have increased dramatically following COVID-19. To take these assays forward to support clinical trial testing a more robust comparison can be achieved if methods are validated, using quality-checked PV and viruses, and the correct statistical analysis is conducted^54,55^. Furthermore, reporting results relative to a standard used in every assay will increase the consistency of results reducing the variability inherent in cell-based methods. Calibration of the in-house standard to the primary calibrant, the WHO International Standard, allows reporting the results in International Units and increases comparability of the data between laboratories globally.

Overall, the system described here outlines an approach to produce bunyavirus PV which can be employed for the R&D studies on a high-priority viral family and to support MCM’s development and assessment. A limitation of the use of PV is that only responses to the glycoprotein of the virus of interest is evaluated. Therefore, their use is beneficial for the investigation of the initial steps of the virus entry and serological assays, only looking at anti-glycoprotein responses. In the context of a rapid response to a pandemic/epidemic and the 100 Days Mission, these bunyavirus PV which can be produced quickly and adapted to incorporate yet unknown closely related glycoproteins constitute an essential tool in response to public health treats, such as Disease X.

## Methods

### Cell lines

Human embryonic kidney 293T clone 17 cells (HEK293T/17; ATCC CRL-11268) were used to produce PV. African green monkey kidney Vero cells (ATCC, CCL-81) and Vero E6 (ECACC 85020206), feline kidney CRFK cells (ATCC CCL-94), human hepatocellular carcinoma-derived Huh7^56^ (JCRB-0403), SW13 (ECACC 87031801) and Chinese hamster ovary CHOK-1 cells (ATCC CCL-61), were used for titration of PVs and neutralisation assays. Cells were maintained at 37 °C in 5% CO_2_ in Dulbecco’s Modified Eagle Medium with high glucose and GlutaMax^TM^-I (DMEM + GlutaMax^TM^-I; Gibco; 61965) except for CHOK-1 cells, which were maintained in Nutrient Mixture Kaighn’s Modification (F-12K Nut Mix 1X, Gibco). Both media were supplemented with 10% heat inactivated foetal calf serum (FBS, Pan Biotech BmgH), 100 unit/mL penicillin, 100 µg/mL streptomycin (pen/strep; Sigma-Aldrich). In addition, G418 disulfate salt solution (1 mg/mL, Sigma-Aldrich) was added to the media for HEK293T/17 cells.

### Bunyavirus GnGc expression plasmids

GnGc sequences of the RVFV (strain ZH548, NC_014396.1) were cloned into the pI.18^57^ using restriction enzymes KpnI and XhoI. The codon optimised (for *Homo Sapiens*) GnGc sequences of RVFV (strain ZH548, NC_014396.1), CCHFV (IbAr10200 strain, NC_005300.2) OROV (train BeAn19991, KP052851.1) and DABV (isolate 14KS25, MG737104.1) were synthesised to include at the 5′-end a Kozak consensus sequence and flanked by KpnI and XhoI enzymatic restriction sites (Eurofins Scientific and Genewiz, Azeta Life Sciences). All the GnGc sequences were cloned into the expression plasmid pI.18 and confirmed by Sanger sequencing.

### Pseudotyped virus production

The production of HIV-1-based PV was performed as previously described^14^. Briefly, 4 × 10^6^ HEK293T/17 cells were seeded in a 10 cm tissue culture dish and incubated at 37 °C and 5% CO_2_ to reach 60–80% confluency after approximately 24 h. After changing the media, cells were co-transfected with 1 µg of HIV-1 *gagpol* plasmid pCMV-Δ8.91^58^, 1.5 µg of pCSFLW^59^ and various amounts of pI.18 expressing the bunyavirus GnGc by adding the plasmid mix to 200 µL of Opti-MEM (Gibco). The plasmid mix was added to the transfection reagent FuGENE HD (Promega) at a ratio of total plasmid DNA to transfection reagent of 1:3 (w/v) or to 60 µL polyethylenimine, branched (PEI, Sigma-Aldrich) transfection reagent. After an incubation of 20 min, the mix was added to the cells drop-wise and incubated at 37 °C and 5% CO_2_. After 16–20 h the cell media was replaced and supernatant containing the PV collected at 48 and 72 h post-transfection. The supernatant was passed through a 0.45 µm membrane filter (Whatman). Ten-fold concentration of the HIV-1-based particles was performed on a 20% sucrose cushion (5 mL for 25 mL of PV-containing supernatant) and spun at 23,000 rpm for 2 h at 4 °C on a SW28 rotor in a Beckman Optima LE-80K Ultracentrifuge. The pellet was then resuspended in 2.5 mL of complete media, aliquoted, and stored at −80 °C

The detailed protocol for the generation of VSV-based bunyavirus PV is available at protocol.io^60^ and illustrated in Supplementary Fig. 3. Briefly, 4 ×106 HEK293T/17 cells were seeded in a 10 cm tissue culture dish and incubated at 37 °C and 5% CO_2_ until they reached 60-80% confluency. Cells were transfected with pI.18 expressing the bunyavirus GnGc in 200 µL of Opti-MEM and mixed with a transfection reagent, Fugene HD at a ratio of 1:3 (w/v) or 60 µL PEI. The mix was incubated for 20 min before adding to the cells. After 24 h, the media was replaced with 5 mL of FCS-free media containing recombinant ΔG-VSV-luciferase virus (Kerafast)^6^ at an MOI of 0.1 and cells were incubated for 2 h at 37 °C and 5% CO_2_. After incubation, cells were gently washed three times with 3 mL of phosphate-buffered saline (PBS) before adding 8 mL of complete media. After 24 h, the supernatant containing the PV was collected, passed through a 0.45 µm membrane filter, aliquoted and stored at −80 °C.

### Pseudotyped virus titration

Target cells were seeded, at least two hours before titration, either 20,000 cells (HIV-1/PV) or 40,000 cells (VSV/PV) per well in a 96-well plate and incubated at 37 °C, 5% CO_2_. Pv-containing supernatant was fivefold serially diluted in complete media in quadruplicate before addition to the cells. After either 24 h (VSV/PV) or 60 h (HIV-1/PV), infected cells supernatant was replaced with 100 µL of a solution containing the luciferase substrate (Bright-Glo® Luciferase assay system, Promega) and phenol free DMEM (Gibco), ratio1:1 (v/v). After an incubation of 5 min, 90 µL were transferred to a white 96 well plate and the relative light unit (RLU) read using a Glomax Navigator microplate luminometer (Promega). The PV titre was calculated as 50% tissue culture infectious dose per mL (TCID_50_/mL) using the Spearman-Kärber formula^61^.

### VSV-based pseudotyped virus neutralisation assay

In a 96-well plate, two-fold serial dilutions of antibodies or human samples were prepared in duplicate and incubated with 100 TCID_50_ of VSV-PV at 37 °C for 1 h. The mixture was added to 4 × 10^4^ cells/well, seeded in a 96-well plate at least two hours earlier, and incubated at 37 °C for 24 h. After the incubation time, the luciferase activity was measured as described above. The percentage of neutralisation was calculated against average RLU values of cells only (100% neutralisation) and PV only (0% neutralisation). The 50% neutralisation titre was calculated using a 4-parameter dose–response inhibition curve against the logarithm of the sample dose/reciprocal dilution using GraphPad Prism v.10.3.1.

### Microneutralisation assay

Target Vero E6 cells were seeded 20,000 cells/well in a 96-well tissue culture plate the day before to reach an 80-90% confluent monolayer. An input of 100 TCID_50_ of RVFV (strain ZH501, kindly provided by the UK Health Security Agency, UKHSA) was incubated with twofold serial dilutions (in triplicate) of the serum samples at 37 °C for 1 h and then added to the target cells and incubated at 37 °C. After 36–40 h the cells were washed in PBS and fixed in 8% formaldehyde in PBS for 1 h at room temperature. After fixation, the cells were permeabilised with 0.2% Triton-X100 for 15 min. After one wash with PBS/0.05% (v/v) Tween-20 (wash buffer), the cells were incubated in blocking buffer (3% skimmed Milk in wash buffer) for 1 h at room temperature. Cells were then incubated with 50 µL/well of the primary antibody anti-RVFV nucleoprotein mouse monoclonal antibody (M977, The Native Antigen Company) diluted 1:1000 in blocking buffer, for 1 h at room temperature. After three washes, the cells were incubated with 50 µL/well of secondary antibody, polyclonal rabbit anti-mouse IgG Horseradish Peroxidase (HRP) conjugated antibody (Jackson ImmunoResearch) diluted 1:1000 in blocking buffer, for 1 h at room temperature. After three washes, the HRP substrate TMB (K-Blue aqueous TMB substrate, Neogen) was added to the cells and the colorimetric reaction was stopped after 5–15 min with 2 N H_2_SO_4_. The intensity of the enzymatic reaction was measured at a spectrophotometer (Fluostar Omega, BMG Labtech), at the optical density (OD) of 450 nm. NT_50_ was calculated as described above.

### Monoclonal antibodies and human samples

Monoclonal neutralising antibody RVFV-144 (mAb144) was kindly provided by Professor James E. Crowe (Vanderbilt Vaccine Centre, USA). The mouse monoclonal antibody anti-CCHFV pre-Gc glycoprotein mAb11E7 was acquired through BEI Resources, USA (NR-40277). The human monoclonal antibody anti-DABV Gn glycoprotein mAbS2A5 was acquired through Absolute Antibody, UK (Ab04620-10.0)^37^. The Oropouche mouse hyperimmune ascitic fluid AF was acquired through ATCC, USA (VR-1228AF)^38^.

The 60 convalescent sera collected in Kenya from individuals with confirmed or suspected exposure to RVFV were provided by Francis Mutuku and Victor Jeza (Technical University of Mombasa) and Bryson Ndenga (Kenya Medical Research Institute). The collection of human samples was approved by the Technical University of Mombasa Research Ethics Committee (TUM ERC EXT/002/2021). Seven convalescent plasma samples from individuals recovered from Crimean-Congo haemorrhagic fever (2015 or 2017 outbreaks) were collected in 2019 by Dr Nazif Elaldi (Sivas Cumhuriyet University, Sivas, Turkey). The collection was approved by the local Research Ethics Committee of the Ankara Numune Education and Research Hospital, Turkey (Protocol # 17–1338)^41^. All donors gave consent, and samples were provided coded and anonymised.

### Western blot

PV-containing supernatant was concentrated through 20% sucrose cushion in PBS and spin at 20,000 *g* for 2 h at 4 °C. The supernatant was removed, and the PV pellets were resuspended in 1/20 volume of RIPA lysis buffer (Thermo Fisher Scientific) and incubated for 1 h on ice. The lysed PV samples were denatured by adding 2X SDS buffer (Thermo Fisher Scientific), DTT (Thermo Fisher Scientific) and boiled for 5 min at 95 °C. The proteins were separated by SDS-PAGE in a 4–20% Tris-glycine gel (Thermo Fisher Scientific), then transferred to a PVDF membrane (Thermo Fisher Scientific) and blocked with 5% milk (Marvel) in 0.05% Tween-20/PBS. Membranes were probed with mouse anti-RVFV Gn monoclonal antibody (1:1000; RVFG11-M, Alpha Diagnostics) or mouse anti-CCHFV Gn monoclonal antibody (1:1000, MAB12327, Native Antigen), and with secondary antibody goat anti-mouse IgG conjugated to HRP (1:5000, Sigma). Both primary and secondary antibodies were incubated for 1 h at room temperature on a shaker. The membranes were incubated for 5 min in ECL solution (Cytiva) and the chemiluminescence signal was then measured using the Chemidoc MP system (Bio-Rad).

### Statistical data analysis

Statistical analyses were performed using GraphPad Prism version 10.3.1. Pearson correlation, Deming regression and Bland-Altman analysis were used to compare the MNA and PVNA results.

## Supplementary information

Supplementary Information

## Data Availability

All data analysed during this study are included in this published article.
