# Supplementary material for: A system to rapidly develop a bunyavirus pseudotyped virus neutralisation assay for pandemic preparedness
Source: NPJ Vaccines. 2025 Nov 6;10:228. doi: 10.1038/s41541-025-01278-8 (PMC12592406; doi:10.1038/s41541-025-01278-8)

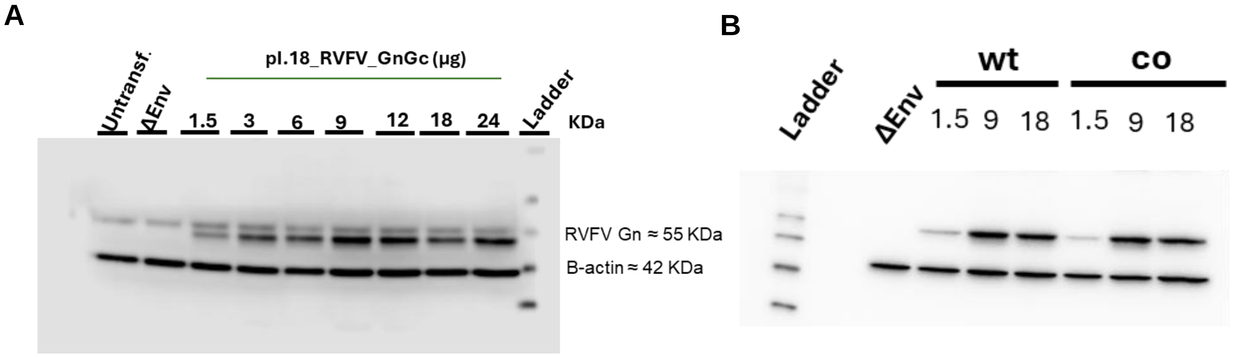

Supplementary Figure 1. Optimisation of RVFV glycoprotein expression. RVFV PV producer HEK293T cells were transfected with increasing amounts of pI.18_GnGc plasmid using Fugene HD transfection reagent (A) or PEI transfection reagent (B). Forty-eight hours post transfection cells were lysed and proteins separated on a SDS-PAGE by electrophoresis. Immunostaining was conducted after dry transfer to PVDF membrane using anti- Anti-RVFV Gn protein, followed by secondary HRP-conjugated anti-mouse Ig or anti-beta actin (Thermo Fisher, MA5-15739-HRP). Expected size of the proteins are indicated.

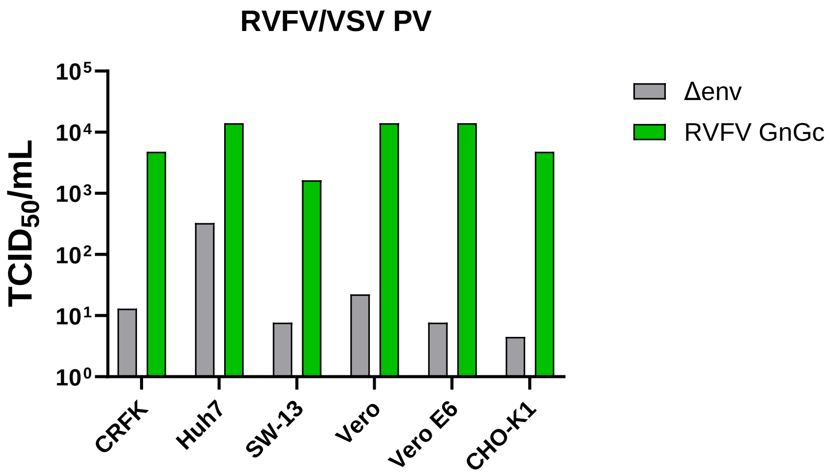

Supplementary Figure 2. Titration of RVFV-PV on different cell lines. Recombinant VSV particles expressing RVFV GnGc were titrated on cell lines commonly used for infectivity assay for RVFV. PV titres are reported based on luciferase activity as $TCID_{50}$/mL. Experiment was conducted once to confirm data in the literature on suitable target cell line for these viruses.

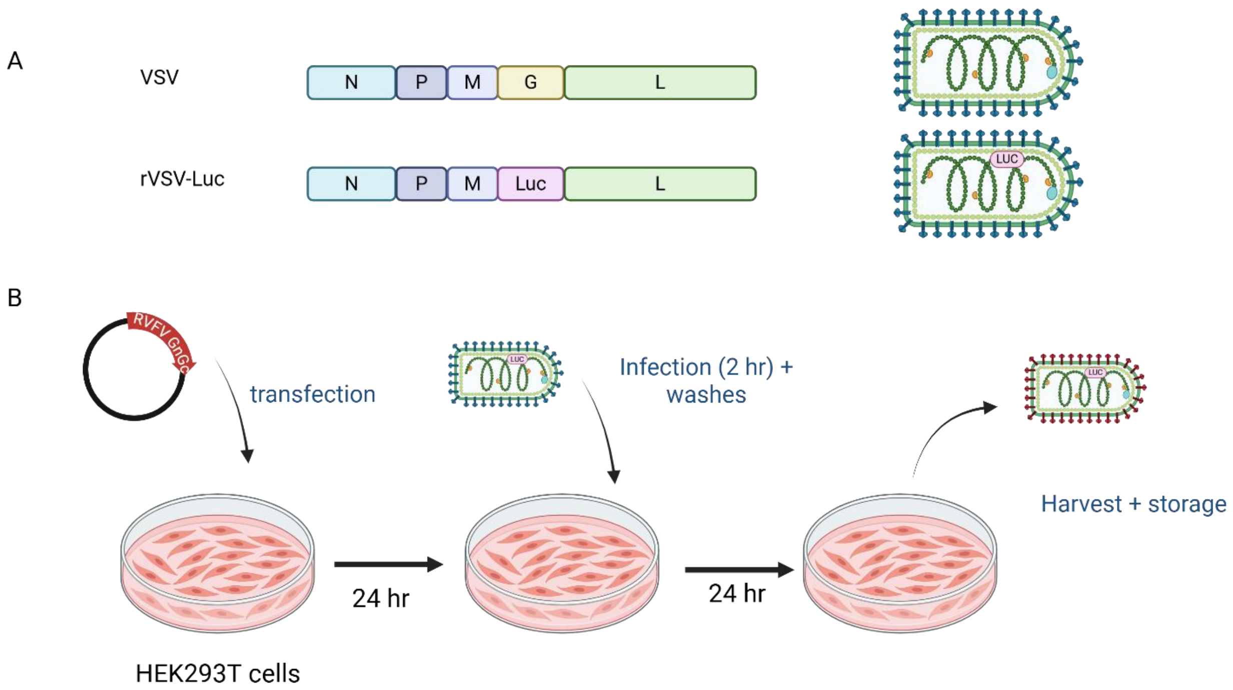

Supplementary Figure 3. Production of VSV-based bunyavirus PV. A) The genome of the recombinant VSV vector has the glycoprotein G gene replaced by the reporter gene firefly luciferase. B) Illustration of the process to generate Rift Valley fever virus GnGc pseudotyped-VSV particles. Created in BioRender. https://BioRender.com/dtvfaen

Original WB for Supplementary Fig 1A

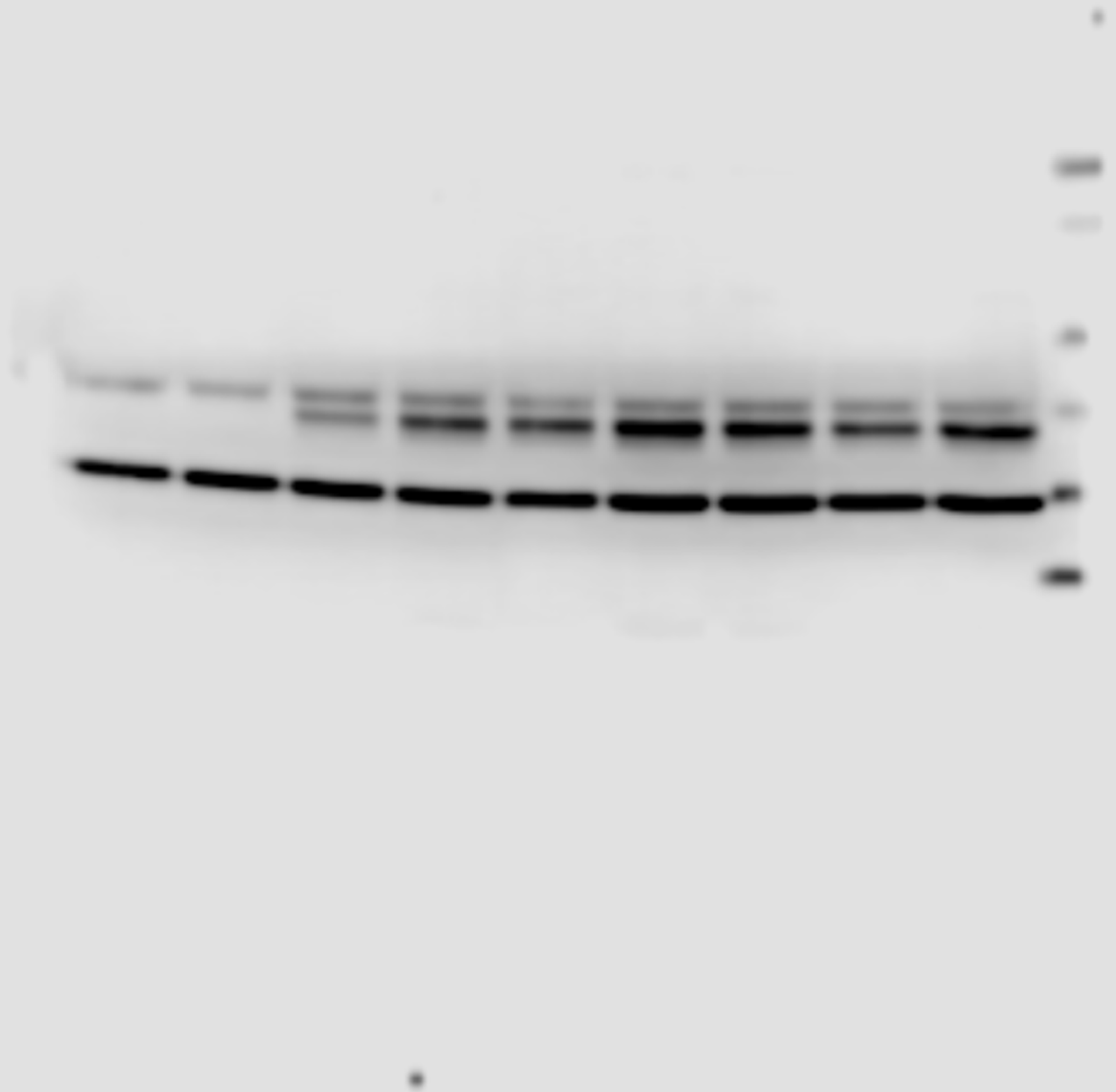

Original WB for Supplementary Fig 1B

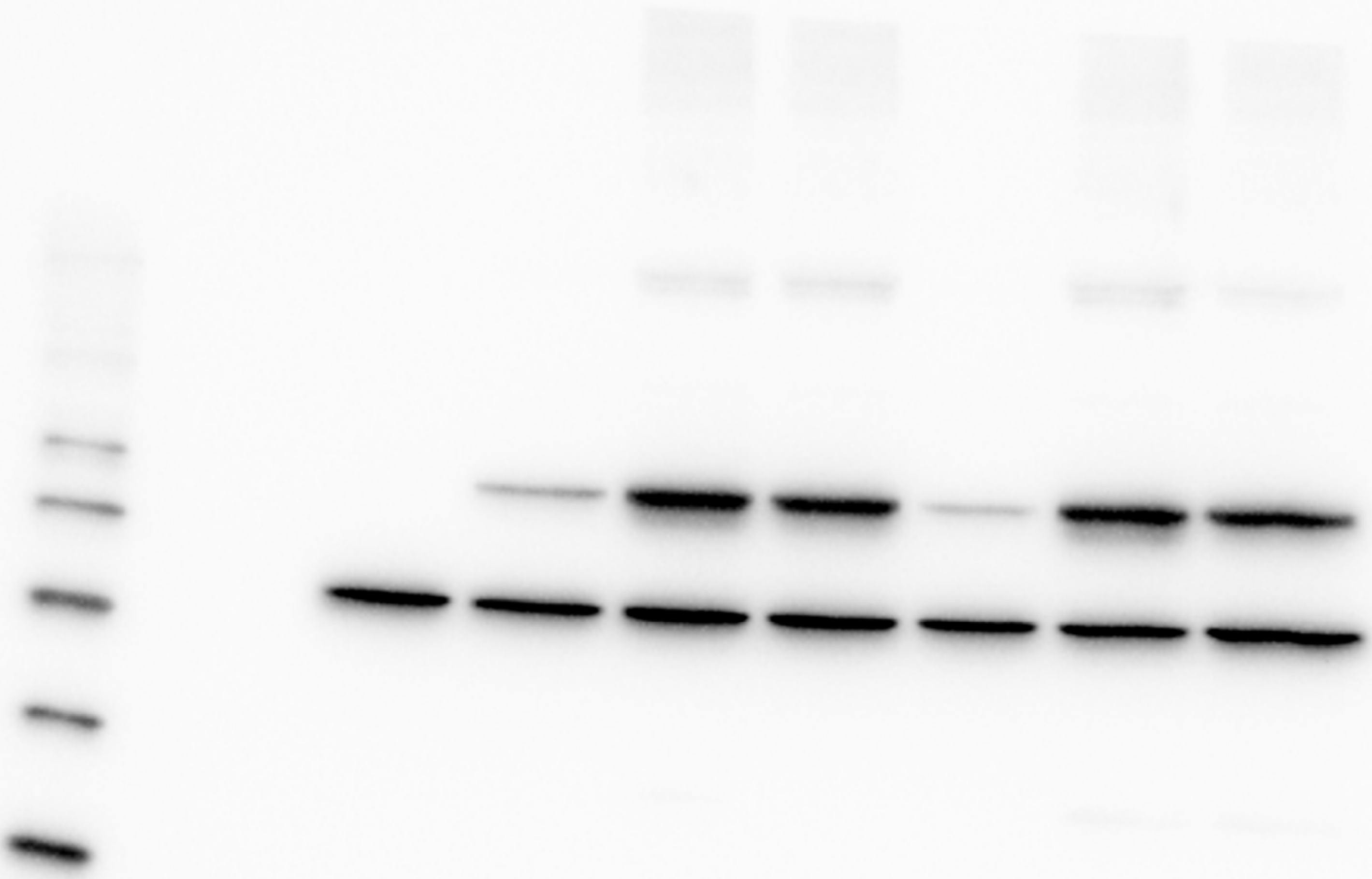

Original WB for Fig 2E

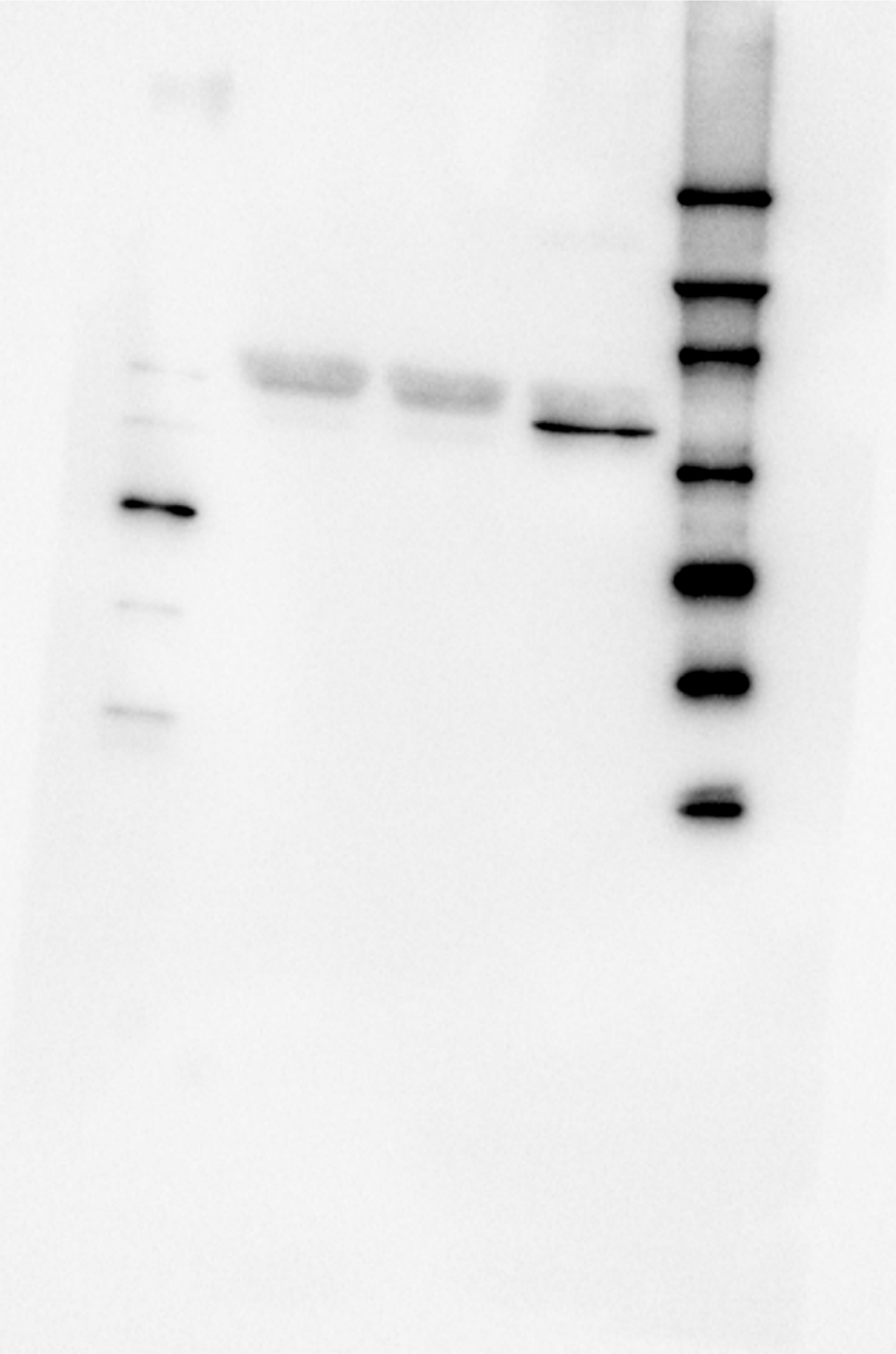

Original WB for Fig 2F

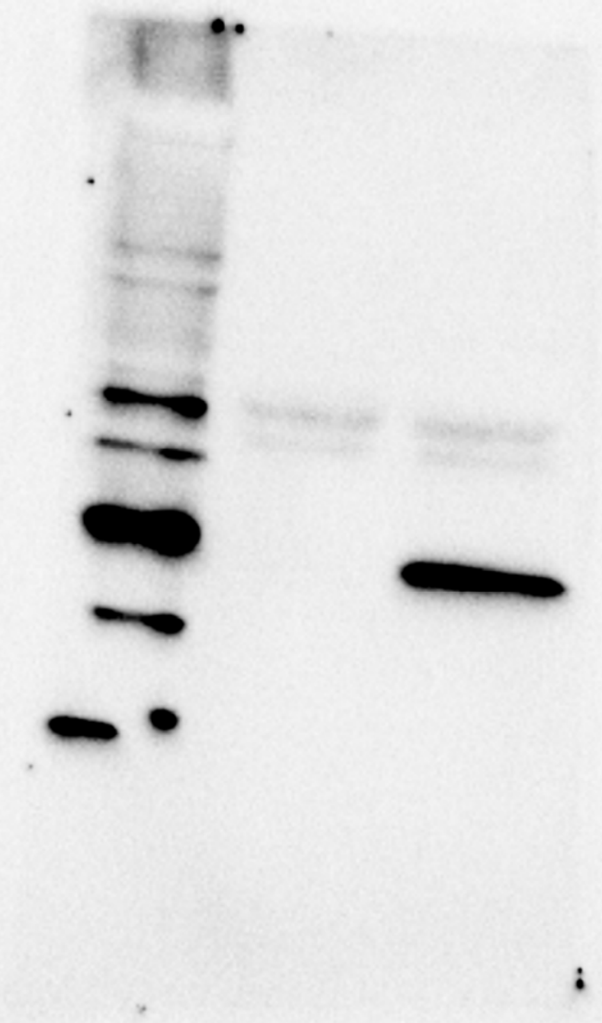

Supplement: Supplementary file 1 — Supplementary Information [file 41541_2025_1278_MOESM1_ESM.pdf]
